# The impact of ultrasound-guided vascular access for catheter ablation of left atrial arrhythmias in a high-volume centre

**DOI:** 10.1007/s10840-024-01779-x

**Published:** 2024-04-04

**Authors:** Amelie Krimphoff, Lukas Urbanek, Stefano Bordignon, David Schaack, Shota Tohoku, Shaojie Chen, K. R. Julian Chun, Boris Schmidt

**Affiliations:** Cardioangiologisches Centrum Bethanien, Agaplesion Markus Krankenhaus, Goethe Universität, Wilhelm-Epstein Str. 4, 60431 Frankfurt/Main, Germany

**Keywords:** Atrial fibrillation, Atrial arrhythmias, Catheter ablation, Ultrasound-guided vascular access, Vascular access complications

## Abstract

**Background:**

Vascular complications are a common occurrence during atrial fibrillation ablation. Observational studies indicate that the utilization of ultrasound (US)-guided puncture may decrease the incidence of vascular complications; however, its routine use is not established in many centres.

**Methods:**

Patients undergoing catheter ablation for atrial fibrillation were included sequentially. All patients receiving US-guided punctures were prospectively enrolled (US group), while patients who underwent the procedure with standard puncture technique served as control group (No-US group). Periprocedural vascular complications requiring intervention within 30 days of the procedure were defined as the primary endpoint.

**Results:**

A total of 599 patients (average age: 69 ± 11 years, 62.9% male) were analysed. The incidence of vascular complications was lower with the US-guided puncture than with the anatomic landmark-guided puncture (14/299 [4.7%] vs. 27/300 [9%], *p* = 0.036). The US-guided vascular access significantly reduced the rate of false aneurysms (3/299 [1%] vs. 12/300 [4%], *p* = 0.019). In addition, the occurrence of arteriovenous fistula (2/299 [0.7%] vs. 4/300 [1.3%], *p* = 0.686) and haematoma requiring treatment (9/299 [3%] vs. 11/300 [3.7%], *p* = 0.655) were also lower in the US group. US-guided puncture did not prolong the procedure time (mean procedure time: 57.48 ± 24.47 min vs. 56.09 ± 23.36 min, *p* = 0.478). Multivariate regression analysis identified female gender (OR 2.079, CI 95% 1.096–3.945, *p* = 0.025) and conventional vascular access (OR 2.079, CI 95% 1.025–3.908, *p* = 0.042) as predictors of vascular complications.

**Conclusions:**

The implementation of US-guided vascular access for left atrial catheter ablation resulted in a significant decrease of the overall vascular complication rate.

## Introduction

Atrial fibrillation (AF) is the most common cardiac rhythm disorder affecting 2 to 4% of adults [1]. In recent decades, there has been a significant increase in the number of AF ablation procedures [2]. Catheter ablation is a well-established treatment option to reduce arrhythmia-related symptoms and improve quality of life [1]. The most frequent complications of AF ablation are related to vascular access, with a reported incidence varying from 2 to 4% according to the latest European Society of Cardiology guideline [1]. Continuous anticoagulation, intraprocedural systemic anticoagulation and the use of multiple large-diameter sheaths during AF ablation increase the risk of bleeding [3]. Conventionally, femoral venous access was achieved by ‘blind’ puncture medial to where the femoral arterial pulse was palpated. However, computer tomography scans showed overlapping femoral artery and vein in two-thirds of patients [4]. To facilitate vascular access, real-time ultrasound (US) guidance is recommended in many medical specialities, including anaesthesiology [5], intensive care [6], nephrology [7] and paediatrics [8]. However, routine use of US-guided vascular access is not established in many electrophysiology centres. Furthermore, a prospective randomised study found no clear advantage of US-guided venous puncture in reducing vascular access complications [9]. The present study investigated the impact of US-guided venous access for left atrial (LA) ablation procedures in a high-volume ablation centre.

## Materials and methods

The study was approved by the local ethics committee (2022–2956-evBO) and complies with the Declaration of Helsinki.

Patients undergoing catheter ablation for LA arrhythmias were enrolled consecutively at a single high-volume ablation centre (1400 AF catheter ablations per year). All patients undergoing US-guided vascular access between August and October 2022 were prospectively enrolled (US group). Patients who underwent the procedure with anatomic landmark-guided puncture between September and November 2021 served as control group (No-US group). Before the investigation had started, anatomic landmark-guided cannulation was the standard puncture technique in our institution. The US-guided venous access was introduced for this study.

### Study participants

All patients over 18 years of age with AF or LA tachycardia were eligible to enter the study. Participants in the US group had to sign the patient informed consent form before enrolment. Planned arterial puncture for the procedure led to exclusion from the study.

### Anticoagulation

Novel oral anticoagulation (NOAC) therapy was minimally interrupted by skipping the morning dose on the day of the procedure and resuming NOAC therapy six hours after the procedure. Uninterrupted vitamin K antagonist therapy with an INR value between 2 and 3 was aimed. In patients naive to oral anticoagulation (OAC), NOAC therapy was started six hours after ablation.

### Ablation procedure

All ablation procedures were performed under deep intravenous sedation using midazolam, fentanyl and propofol. The right femoral vein was used to gain venous access. After palpation of the arterial pulse and application of local anaesthesia, the physician obtained vascular access with the Seldinger technique. Depending on the ablation modality, inserted sheaths differed in size and number. Standard radiofrequency (RF) ablation required three sheaths with an inner diameter (ID) of 8,5 French (Fr). Standard single-shot ablation required one 8 Fr and one large-diameter sheath (12–13.8 Fr ID). After venous access, intravenous unfractionated heparin was administered according to the body weight to maintain an activated clotting time of at least 300 s. No protamine was administered at the end of the ablation. The catheters and sheaths were removed and the femoral vein was routinely sealed with a Z-suture. Pressure tape was rarely applied according to the operator's preference, particularly in cases of immediate post-procedural bleeding, inadvertent arterial puncture, in extremely thin patients or additionally in the case of insufficient Z-suture.

This represents the standard workflow at our institution. The procedure and peri-procedural patient management were identical in both groups.

### US-guided puncture

**Fig. 1:**
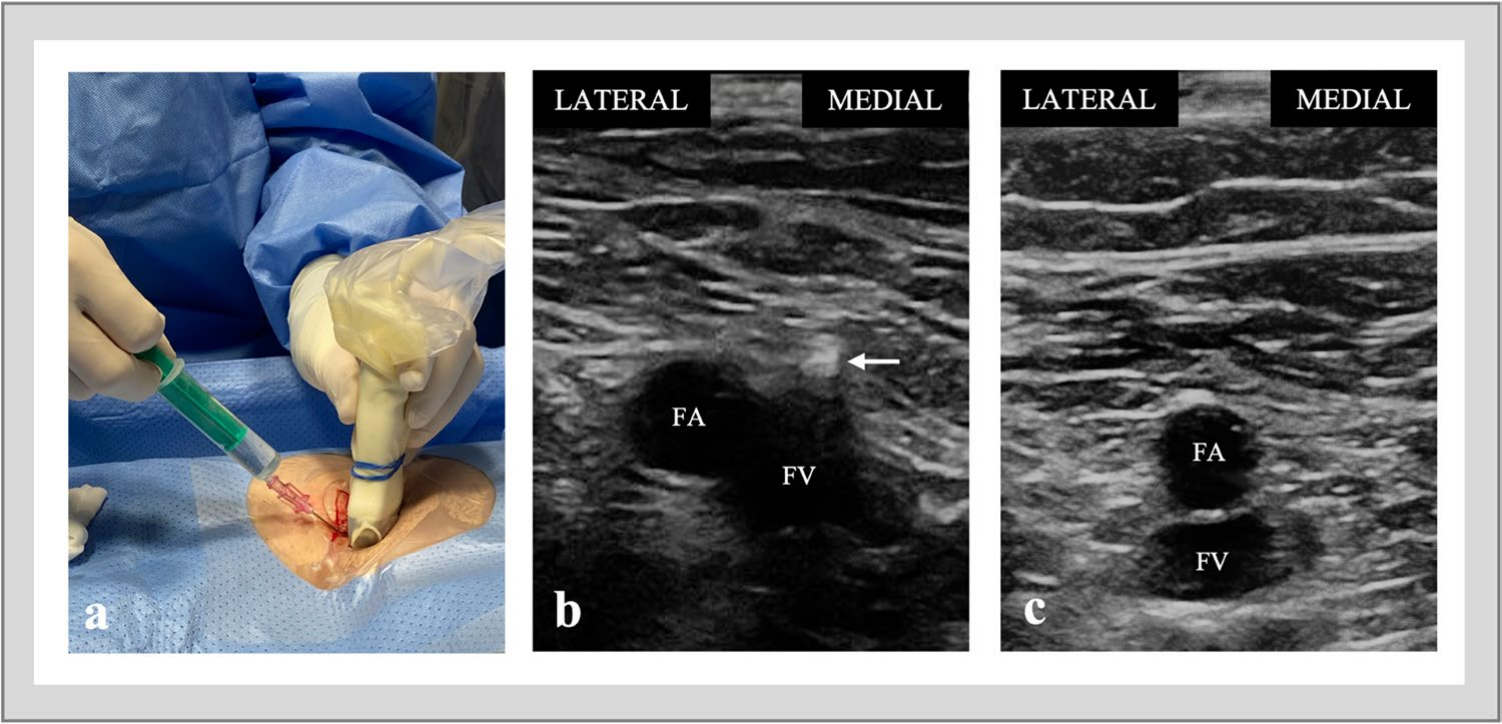
US-guided venous access. **a** Two-handed puncture technique. **b** The optimal puncture site with the femoral vein situated medial to the femoral artery and visualisation of the needle just before entering the vein (white arrow). **c** The suboptimal puncture site with the femoral vein located inferior to the femoral artery. FA, femoral artery; FV, femoral vein

A linear array probe was covered with a sterile sheath for US-guided puncture. The probe was positioned below the inguinal ligament at a 90-degree angle to the femoral vein, displaying the vessels in short axis (Fig. 1a). The venous image was placed in the middle of the screen. The femoral vein is a compressible structure typically located medial to the non-compressible femoral artery. To avoid an inadvertent arterial puncture, the artery and vein should be side by side, not on top of each other (Fig. 1b, c). The needle tip or at least the tissue moving should be visible when penetrating the venous wall.

Before starting the study, all physicians performed at least ten roll-in cases with US-guided vascular access. Training cases were excluded from the analysis. All physicians were experienced in the use of anatomic landmark-guide puncture.

### Endpoints

The primary endpoints were vascular complications, including pseudoaneurysm, arteriovenous (AV) fistula or haematoma requiring treatment such as transfusion, surgery, prolonged mechanical compression, analgesics or extended hospitalisation, within 30 days. Prolonged compression is defined as secondary haemorrhage on the ward requiring continued compression after primary Z-suture or pressure tape. Major vascular complications encompassed complications that resulted in extended hospitalisation, readmission or required intervention such as surgery or transfusion. When participants were diagnosed with multiple complications, only the most relevant complication was analysed (vascular complications relevance: pseudoaneurysm > AV fistula > haematoma).

Secondary endpoints were the procedure time and days of hospitalisation.

### Post-procedural management and follow-up

OAC was resumed six hours after ablation. Before discharge, patients underwent daily clinical examinations, including inspection, palpation and auscultation of the puncture side. A colour duplex sonography was performed in the event of abnormalities in the clinical examination. All examination results were documented in the electronic patient record. Post-procedural inpatient monitoring was planned for two nights. After discharge, all patients were instructed to return to our outpatient clinic in case of any vascular complications. For a period of 30 days after hospital discharge, each clinical visit for a vascular complication was followed up.

### Statistic

Continuous variables were presented as mean and standard deviation or median and interquartile range. A two-sided *t* test for independent samples was used to compare continuous variables with normal distribution. In case of variance heterogeneity, the Mann–Whitney *U* test was performed. Categorical variables were summarised as frequencies and percentages and compared with the Chi-square or Fischer’s exact test.

Binary logistic regression, expressed as odds ratio (OR) with 95% confidence interval (CI), was performed to identify independent predictors of vascular complications. Considering the interaction of various risk factors, a multivariable model was constructed including the following covariates: Body mass index (BMI), female gender, RF ablation and conventional vascular access. A* p*-value < 0.05 was considered to indicate statistical significance. Statistical analysis was performed by using the SPSS statistical package, version 29.0.

## Results

### Patients

Overall, 600 patients were included, 300 per group. One patient in the US group was excluded due to an intended arterial puncture on the venous access site, leaving 299 patients for analysis. In the US group, one patient developed a haematoma after the initial puncture, which made it impossible to identify the vessels on the US image. Therefore, the last two punctures were performed using anatomic landmark-guided puncture.

### Baseline characteristics

Demographic and clinical baseline characteristics were well-matched between the two groups, except for a higher prevalence of pre-existing diabetes mellitus in the control group (14.7% vs. 8.7%, *p* = 0.023) and higher INR values (1.2 [1.09–1.36]) vs. 1.23 [1.1–1.5], *p* = 0.043) in the US group. Detailed patient characteristics are summarised in Table 1. Most patients were male (62.9%), on average 69 years old, and the vast majority of patients were receiving NOACs (85%).

**Table 1:** Baseline characteristics

	Total (*n* = 599)	Conventional (*n* = 300)	US (*n* = 299)	*p*-value
Age (years)	68.45 ± 10.73	68.8 ± 10.5	68.1 ± 10.97	0.428
Female	222 (37.1%)	112 (37.3%)	110 (36.7%)	0.89
Body mass index (kg/m^2^)	28.07 ± 7.89	27.81 ± 5.31	28.32 ± 9.82	0.428
Arterial hypertension	427 (71.3%)	219 (73%)	208 (69.6%)	0.353
Diabetes mellitus	70 (11.7%)	44 (14.7%)	26 (8.7%)	0.023 *
History of stroke	37 (6.2%)	18 (6%)	19 (6.4%)	0.857
Heart failure	22 (3.7%)	12 (4%)	10 (3.3%)	0.670
CAD	92 (15.4%)	45 (15%)	47 (15.7%)	0.807
GFR (ml/min)	85.1 ± 34.67	83.86 ± 34.91	86.33 ± 34.46	0.385
CHA2DS2VASc score	2 (1–3)	2 (1–3)	2 (1–3)	0.252
INR	1.21 (1.1–1.42)	1.2 (1.09–1.36)	1.23 (1.1–1.5)	0.043*
First procedure	424 (71%)	201 (67.4%)	223 (74.6%)	0.055
New oral anticoagulants	509 (85%)	257 (85.7%)	252 (84.3%)	0.635
Vitamin K antagonists	15 (2.7%)	8 (2.7%)	7 (2.3%)	0.789
Antiplatelet drug	34 (5.3%)	14 (4.7%)	20 (6.7%)	0.285

Values are presented as mean ± SD, median and interquartile range or as n (%). * Statistically significant results, *p* < 0.05 *CAD*, coronary artery disease; *GFR*, glomerular filtration rate; *INR*, international normalized ratio; *US*, ultrasound

In 51.4% and 48.6% of patients, point-by-point RF and single-shot ablation were performed, respectively (*p* = 0.513). For single-shot modalities, pulsed field ablation (41.9%), cryoballoon (39.9%), RF balloon (13.7%) and laserballoon (4.5%) were used. Vascular access was most frequently closed with a Z-suture (No-US: 93% vs. US: 96.3%, *p* = 0.071). In a minority of cases, pressure tape was used to seal the access site (5.3% vs. 3.7%, *p* = 0.329), including six patients in whom Z-suture was initially used for vascular occlusion. In the No-US group, a vascular closure device was used in five patients to close vascular access.

### Study endpoints

The primary outcome measure—the rate of vascular complications within 30 days of the ablation procedure—is shown in Table 2. US-guided access was associated with a significant reduction in the overall incidence of vascular complications (4.7% vs. 9%, *p* = 0.036) and the rate of false aneurysms (1% vs. 4%, *p* = 0.019). In addition, the incidence of AV fistula (0.7% vs. 1.3%, *p* = 0.686) and haematoma requiring treatment (3% vs. 3.7%, *p* = 0.655) was numerically lower in the US group.

**Table 2:** Primary endpoints

	Total (*n* = 599)	Conventional (*n* = 300)	US (*n* = 299)	*p*-value
Total	41 (6.8%)	27 (9%)	14 (4.7%)	0.036 *
Pseudoaneurysm	15 (2.5%)	12 (4%)	3 (1%)	0.019 *
AV fistula	6 (1%)	4 (1.3%)	2 (0.7%)	0.686
Haematoma ^a^	20 (3.3%)	11 (3.7%)	9 (3%)	0.655

Values are presented as n (%). * Statistically significant results, *p* < 0.05. ^a^ Haematoma including transfusion, surgery, prolonged mechanical compression, analgesics, or extended hospitalisation.* AV*, arteriovenous; *US*, ultrasound

US-guided vascular access must be performed in 24 patients to prevent one vascular complication. The rate of major vascular complications was significantly reduced with US-guided venous access (1% vs. 6.3%, *p* =  < 0.001).

**Fig. 2:**
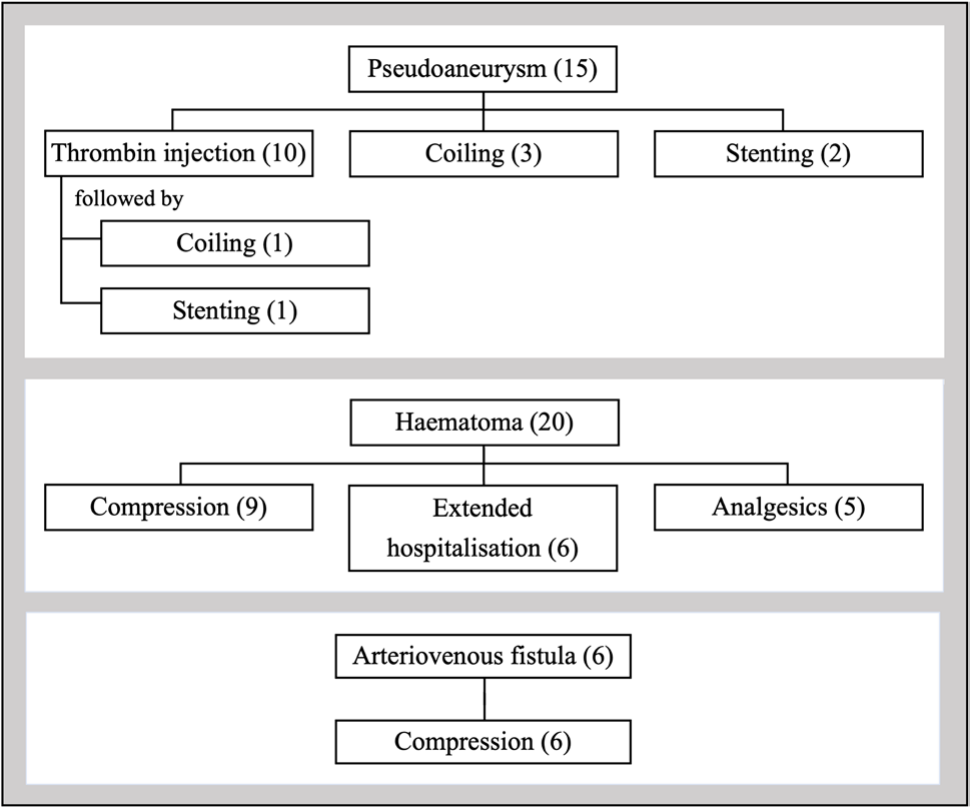
Interventional management of complications

The way the different types of complications were managed is illustrated in Fig. 2. Interventional management was required in all 15 patients diagnosed with pseudoaneurysm. The six patients with AV fistulas were treated with pressure tape. In the case of a haematoma, interventions included prolonged compression, prolonged hospital stay and analgesics for groin pain. Pressure tape was consistently applied for prolonged compression, except in one case using a femoral compression system called ‘FemoStop’. Transfusions and surgery were required only in two patients with a pseudoaneurysm, who were finally treated with coil embolisation.

Secondary endpoint analysis showed that days of hospital stay were significantly lower in the US group (2.02 ± 0.61 vs. 2.18 ± 1.22, *p* = 0.043). Moreover, US guidance did not prolong the procedure time (57.48 ± 24.47 min vs. 56.09 ± 23.36 min, *p* = 0.478).

### Predictors of vascular complication

As demonstrated in Table 3, univariate regression analysis identified female gender (OR 2.073, CI 95% 1.095–3.922, *p* = 0.025) and conventional vascular access (OR 2.013, CI 95% 1.034–3.921, *p* = 0.04) as predictors of vascular complications. On multivariate regression analysis, female gender (OR 2.079, CI 95% 1.096–3.945, *p* = 0.025) and conventional vascular access (OR 2.079, CI 95% 1.025–3.908, *p* = 0.042) were identified as independent predictors of vascular complications.

**Table 3:** Univariate and multivariate regression analysis for predictors of vascular complications

	Univariate regression		Multivariate regression	
	Odds ratio (95% confidence interval)	*p*-value	Odds ratio (95% confidence interval)	*p*-value
Age (years)	1.004 (0.974 – 1.034)	0.814		
Female (vs. male)	2.073 (1.095 – 3.922)	0.025 *	2.079 (1.096 – 3.945)	0.025 *
Body mass index (kg/m^2^)	0.992 (0.944 – 1.043)	0.753	0.995 (0.946 – 1.046)	0.844
Paroxysmal AF (vs. persistent)	1.573 (0.816 – 3.034)	0.176		
Redo procedure (vs. first procedure)	1.054 (0.523 – 0.124)	0.883		
Conventional access	2.013 (1.034 – 3.921)	0.04 *	2.079 (1.025 – 3.908)	0.042 *
Procedure time (min)	1.000 (0.987 – 1.014)	0.969		
RF (vs. single shot)	0.893 (0.473 – 1.684)	0.726	0.904 (0.477 – 1.715)	0.758

* Statistically significant results, *p* < 0.05 *RF*, radiofrequency ablation; *US*, ultrasound

## Discussion

Vascular access complications remain the most common complication of AF ablation procedures [10, 11]. Although most often not life-threatening, it may be associated with increased consumption of health care resources including interventional treatment, surgery, and prolonged hospitalisation.

A most recent study suggested that the use of US did not significantly reduce the number of procedure-related complications for experienced operators [9]. However, the study was halted prematurely due to an unexpectedly low complication rate.

The current study found that the implementation of US significantly reduced the incidence of complications by approximately 50%. Expectedly, female gender was identified as another independent predictor of access site complications, doubling the risk for a complication.

### Incidence of primary endpoints

Heterogeneous definitions contribute to the wide variations of reported incidences of vascular complications, which range from 2 to 14% [12, 13]. This study considered a wider range of endpoints, including haematomas resolved with compression or analgesics and conservatively treated AV fistulas, which explains a relatively high overall complication rate of 6.8%. Additionally, all data were collected within a 30-day follow-up period, whereas numerous register studies solely analysed inpatient events when reporting complication rates. However, the lack of routine 30-day follow-up may have underestimated the rate of vascular complications.

### Applicability of results

In recent studies, female gender was identified as a risk factor for major complications in catheter ablation [14, 15]. Dugo et al. speculated that the smaller diameter of the vessels and the closer proximity of the femoral vessels in women may be a cause of the gender-related differences in vascular complications [16]. However, the advantages of using ultrasound for all patients and ablation strategies can still be inferred beyond just women. The current study confirmed previous findings, including a meta-analysis, that BMI is not an independent predictor of vascular access complications [17, 18]. Nonetheless, conventional venous access guided by femoral pulse palpation may be particularly challenging in obese patients. US guidance provides a sound solution to decrease the incidences of unsuccessful attempts, inadvertent arterial puncture and vascular complications [19, 20].

The number and size of the sheaths used for AF catheter ablation did not seem to be a significant factor in the incidence of groin complications. This confirms recent randomised studies comparing irrigated RF current ablation with single-shot devices, which are usually associated with large bore sheath access. In the Fire and Ice Trial, there was a non-significant difference in groin complications between the RF (4.3%) and the cryoballoon (1.9%) groups (*p* = 0.09). Similarly, in the laser versus RF for persistent AF study, no significant differences were found [21, 22].

Data on the risks associated with the placement of large-diameter venous sheats are provided by interventional valve reconstructions using 24 Fr sheats. There were higher rates of major vascular complications in mitral (3.5%) and tricuspid (11%) valve reconstruction [23, 24]. However, comparing the risks associated with AF ablation procedures is challenging due to continuous anticoagulation, intraprocedural systemic anticoagulation, and the use of multiple sheats during AF ablation.

### Complication management

Pseudoaneurysm was the most common complication after conventional venous puncture, leading to interventional treatment in all cases. These interventions (i.e. thrombin injection, coil embolisation and stent implantation) are associated with additional risks for complications [25]. In addition, complication management also contributes to higher healthcare expenditures resulting in a worse cost–benefit ratio of AF catheter ablation [26]. Bode and co-workers conducted an analysis that revealed a rise of costs from €139.54 to €153.31 per vascular complication, primarily attributable to the occurrence of pseudoaneurysm and longer hospital stay. In this study and further investigations, these parameters were reduced in the US group [27]. Therefore, implementing US-guided vascular access has a positive economic impact by reducing the incidence of vascular complications.

Uninterrupted OAC substantially reduces the risk for peri-procedural stroke [28]. Research has shown that the risk of stroke after ablation increases within the initial 30 days [29]. In the event of bleeding, the cessation of OAC often increases the risk of stroke. This highlights the importance of secure vascular access to enable uninterrupted stroke prophylaxis.

### Impact on work-flow with focus on same-day discharge

Work-flow optimisation is key to treat a growing number of patients effectively. Implementation of US-guided venous access is associated with minimal added expense for a sterile cover and should be available at almost all hospitals. Additionally, it does not lead to prolonged procedure times and has been demonstrated to actually shorten puncture time in other studies [17, 30]. In the study referred to above, the use of US resulted in lower puncture time, as well as a reduction in the number of inadvertent arterial punctures and puncture attempts. These findings were consistent for both trainees and expert operators, indicating that the introduction of US-guided vascular access has a short learning curve [31].

Recently, several observational studies suggested that AF ablation procedures based on a same-day discharge strategy are feasible and safe [32]. The absence of vascular access complications plays a crucial role in this regard. It seems that in addition to a safe venipuncture, a vascular closure system can contribute to excellent clinical outcomes [33, 34]. This also translated into improved patient reported outcomes and reduced clinical costs [35].

### Limitations

The main limitation was the non-randomised study design and the retrospectively collected data of the control group. To minimise the selection bias, all patients were enrolled consecutively. However, this study design resulted in a difference between the groups concerning the prevalence of diabetes mellitus and in the INR levels. Although there was a significant difference in INR values in the analysis, this slight dissimilarity is not clinically relevant and instead would increase the risk of bleeding in the US group. The effect of diabetes mellitus the rate of vascular complications was investigated in similar studies and diabetes mellitus was not an independent predictor of vascular complications [30, 36]. Therefore, it is unlikely that this is the reason for the differences in vascular complications. Moreover, there were group differences in relation to the operator who carried out the venous access.All venous punctures in the No-US group were performed by experienced operators, while three additional fellows were involved in the US group. Although more fellows performed vascular access in the US group, the rate of access-related complications could be reduced. In addition, all operators in this study were unfamiliar with US-guided venous puncture at the beginning of the analysis. This suggests that US-guided vascular access is easy to learn, even for inexperienced operators. Overall, there is a risk of confounding, as in all observational studies, which limits the validity of the conclusions. Independent physicians performed the follow-up and duplex US in case of complications to minimise observer bias. At least, conducting this investigation in a high-volume centre with a complex study population carries a risk of referral bias.

## Conclusion

In a high-volume AF ablation centre, the introduction of US-guided venous access did result in a significant reduction of venous access site complications. An adequately powered randomised multi-centre trial is urgently needed.

## Data Availability

The raw data will be shared with other researchers on reasonable request.
